# Examining study participants’ decision-making and ethics-related experiences in a dietary community randomized controlled trial in Malawi

**DOI:** 10.1186/s12910-021-00729-w

**Published:** 2021-12-03

**Authors:** Limbanazo Matandika, Kate Millar, Eric Umar, Edward Joy, Gabriella Chiutsi-Phiri, Joseph Mfutso-Bengo

**Affiliations:** 1 Center for Bioethics in Eastern and Southern Africa, College of Medicine, University of Malawi, Private Bag 360, Blantyre, Malawi; 2 Centre for Applied Bioethics, Schools of Biosciences and Veterinary Medicine and Science, University of Nottingham, Sutton Bonington Campus, Loughborough, LE12 5RD UK; 3 Health Systems and Policy Department, College of Medicine, University of Malawi, Private Bag 360, Blantyre, Malawi; 4 Faculty of Epidemiology and Population Health, London School of Hygiene & Tropical Medicine, Keppel Street, London, WC1E 7HT UK; 5 Faculty of Life Science and Natural Resources, Natural Resources College, Lilongwe University of Agriculture and Natural Resources, P.O Box 143, Lilongwe, Malawi

**Keywords:** Ethical roles, Embedded ethics, Informed consent, Self-efficacy, Voluntarily, Rumours, Community randomized control trial, Malawi

## Abstract

**Background:**

The participant recruitment process is a key ethical pivot point when conducting robust research. There is a need to continuously review and improve recruitment processes in research trials and to build fair and effective partnerships between researchers and participants as an important core element in ensuring the ethical delivery of high-quality research. When participants make a fair, informed, and voluntary decision to enroll in a study, they agree to fulfill their roles. However, supporting study participants to fulfill study requirements is an important ethical obligation for researchers, yet evidenced as challenging to achieve. This paper reports on participants’ motivations to volunteer and remain part of a dietary study conducted in Kasungu District, Malawi.

**Methods:**

We conducted twenty in-depth interviews (with chiefs, religious leaders, trial participants, and health surveillance assistants), five systematic ethnographic observations, and fourteen focus group discussions with trial participants and their partners. Interviews were audio-recorded and transcribed verbatim. We used a grounded theory methodology to analyse data that included coding, detailed memo writing, and data interpretation.

**Findings:**

The findings reveal that many participants had concerns during the trial. Thematically, experiences included anxieties, mistrust of researchers, rumours, fears of exploitation, and misconceptions. Anonymous concerns collected from the participants were reported to the trial team which enabled the researchers to appropriately support participants. Despite initial concerns, participants described being supported and expressed motivation to take up their role.

**Conclusion:**

These findings highlight a diverse map of multiple notions of what is ethically relevant and what can impact participation and retention within a study. The study has revealed how embedding a responsive approach to address participants’ concerns and ethical issues can support trust relationships. We argue for the need to employ embedded ethics strategies that enhance informed consent, focus on participants’ needs and positive experiences, and support researchers to fulfill their roles. This work highlights the need for research ethics committees to focus on the risks of undue influence and prevent exploitation especially in settings with a high asymmetry in resources and power between researcher and participant groups.

*Trial Registration*: The Addressing Hidden Hunger with Agronomy (Malawi) trial was registered on 5th March 2019 (ISCRTN85899451).

## Background

The need to improve recruitment processes in research trials and to build fair and effective partnerships between researchers and participants is important for ensuring the ethical delivery of high-quality research [1]. Clear ethical responsibilities to participants, as mediated through the application of ethical principles such as autonomy which are operationalized in Research Ethics Committee (REC) reviews, are set out in the recruitment processes, involving community engagement and informed consent procedures [2]. This makes the recruitment process a key ethical pivot point and as such operationalizing these ethical principles has focused on researchers and research institutions identifying, analyzing, and responding to potential issues [3], and exploring perceptions of study participants, their experiences across the research cycle and how they see or frame their role as participants. Previous studies have explored trial participants’ experiences in a clinical trial setting and the motivation of participants to take part in research [4, 5], however, there appears to be limited work that explores participant’s experiences in nutrition community trials and how they can be supported to fulfill the research study requirements if they wish to remain in a study. Supporting participants is an ethical obligation for researchers when participants make an informed decision to enroll in a study [6, 7], researchers play an active role in supporting and protecting participants as they continuously and freely commit to a study. As researchers actively seek to meet their responsibilities and improve participants’ experiences [8–11], it is important to understand participants’ perceptions of different approaches used to support their voluntary participation.

In terms of conducting ethically robust and reproducible research, including dietary studies in the field of nutrition, accomplishing recruitment goals and supporting adherence to participant protocols is critical [9, 12]. A better understanding of participants’ experiences not only supports an embedded process of respect and enhances ethical responsiveness to their experiences, but there is also an important potential short-term benefit in terms of participant retention within a study. In addition, greater understanding and respect can foster longer-term social benefits of direct and indirect support for research [13], especially for dietary nutrition studies that require participants’ commitment and adherence to a dietary regime. Statistically robust evaluation of trial data relies on sufficient retention of trial participants [9, 14]. The importance of maintaining and improving on high ethical standards, particularly in terms of informed consent processes, in Randomized Controlled Trials (RCTs) is widely recognized. Poor retention of study participants is also known to have a significant impact on the scientific viability of RCTs [15], hence poor adherence to the requirements of clinical trials can undermine the value of the research [15]. Retaining and recruiting participants in clinical research remains a challenge across Africa and globally [16–19]. Several behavioral studies on dietary interventions have reported challenges with the recruitment and retention of study participants [14, 20]. In dietary studies, study participants must be willing to adhere to study requirements that may include changes in social interactions and roles. For example, participants may be advised to only eat food provided by the researchers [21], and not share designated food with others. However, challenges faced by participants in dietary intervention trials have been reported and these include safety concerns of the interventions, complexity of trial designs, lack of support from family and friends [17, 18], lack of knowledge coupled with rumours and misconceptions about research studies [22–24]. These issues can affect willingness to initially participate or remain within trials and can represent a significant burden for participants, which needs to be identified and managed for the benefit of participants’ wellbeing. In addition, existing evidence demonstrates that the retention of participants affects the delivery of robust results or trials, it is therefore essential for researchers to support participants and to facilitate positive experiences which in turn affects retention and adherence to study protocols.

### Supporting study participants to express concerns and fulfill responsibilities in clinical research

When participants make an informed choice to enroll in a study, they voluntarily agree to fulfill study responsibilities [25]. During clinical research, research staff can support participants in various ways [25], and these include an ethically robust informed consent process that underscores the essence of participants freely committing to study requirements and seeking clarification where they lack understanding [2]. Research staff can also support participants through enhanced interaction as well as highlighting barriers to study participation [25].

Accordingly, many issues can influence study participants’ commitment to research protocols [10]. These include researchers being unfamiliar with the research protocol and failing to fully inform study participants about their roles and responsibilities. They also include researchers’ inability to effectively answer questions or provide informational needs of study participants [26] or to be available and willing to respond to participants’ questions and needs through the life of the study [21]. Participants’ willingness to remain in a study can also be affected by others around them [10].

Although studies have been conducted to examine participants’ experiences and factors that influence study retention, Malawi is one country in sub-Saharan Africa where there is a dearth of empirical evidence on the experiences and challenges that study participants encounter when they consent to take part in intervention studies, including agriculture, nutrition, and health community trials. Importantly there is little published material on how researchers can fulfill their responsibilities and support participants when taking part in these studies [27].

### The alleviating hidden hunger with agronomy trial

To enhance our understanding, this study examined the experiences of participants of the Alleviating Hidden Hunger with Agronomy (AHHA) trial. This trial was part of a larger GeoNutrition project implemented by, *inter alia,* the Lilongwe University of Natural Resources (LUANAR), the London School of Hygiene & Tropical Medicine (LSHTM), the University of Malawi, College of Medicine, (CoM), and the University of Nottingham. The overall aim of the AHHA trial was to determine the efficacy of addressing selenium deficiencies in Malawi through the consumption of agronomically-biofortified maize flour [12]. The AHHA trial was a double-blind, randomized, controlled trial conducted in a community setting in rural Kasungu District, Central Region, Malawi. The trial enrolled healthy non-pregnant women of reproductive age (20–45 years old) and at least one healthy school-aged child (5–10 years old) in permanent residence. Participating households were provided with free maize flour, with quantities sufficient for all household members (the equivalent of 330 g per person per day), and distributions were made every two weeks. To implement good practice approaches in the trial design and implementation, a formative study with community groups was conducted approximately 12 months before the intervention, to identify the feasibility of the trial and any issues that may arise in its delivery [28]. The AHHA study protocol was reported according to Standard Protocol Items: Recommendation for Intervention Trials (SPIRIT) guidelines and the protocol paper was published [21].

### The role of participants in the AHHA study

Potential participants in the AHHA trial were asked to (1) receive and consume assigned maize flour in place of their maize flour for a 12-week study period, (2) provide a blood sample during the baseline and then endline surveys of the trial, (3) ensure they do not share the flour with others and (4) report any adverse events relating to their physical health or social wellbeing. Baseline blood samples were collected two weeks before commencement of the flour distribution process, and endline blood samples were collected after approximately nine weeks of exposure to the intervention. All trial participants were encouraged to attend trial education sessions that were organized by the TT. Households were visited by the Trial Team (TT) once every fortnight to monitor flour consumption compliance. It should be noted that households residing in the trial village but not participating in the trial were offered free maize flour (not biofortified) whether they volunteered to take part in the trial or not. This was done to address potential issues of social inequity within the communities, potential feelings of jealousy or stigma and to reduce the view of receiving flour as an incentive or compulsion to take part, as well as to reduce the likelihood that participating households would share their flour with others.

### The empirical ethics study

This empirical ethics study assessed various complex phenomena by exploring evolving experiences of participants as the AHHA trial rolled out [19]. To do this several research questions informed this work and the analysis of the data presented here:

1. What were the participants’ experiences in the community randomized controlled trial?
2. What did the study participants value in their relationship with the study team?
3. What were the motivators for participants’ adherence to study protocols?
4. What elements of the informed consent approach enhanced participants’ comprehension of the study requirements?

The research reported here included the continuous process of listening to and consulting participants using qualitative research methods, facilitating an ‘emic’ perspective of trial participation [29]. Furthermore, an embedded real-time research ethics approach (RTREA) was intended to support the implementation of the functional and ethically sound strategies that could be applied to support the participants and the TT, which in turn provided an opportunity to address challenges and support participants as they took up their role in the trial.

## Methods

**Fig. 1:**
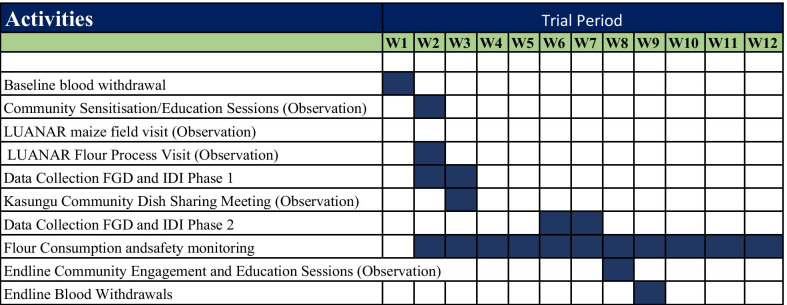
Data collection time points and study activities (weeks 1–12)

Study participants’ experiences were collected by using a range of qualitative methods conducted at different time points across the AHHA trial. Data collection was conducted over seven months from April to October 2019 and data were collected in two phases. Pre-trial data from various activities was collected between April–July whilst data collection during the trial implementation period was collected from July–September, over 12 weeks (Fig. 1). This study was carried out by a bioethics team (BT) that was embedded in the AHHA trial set-up and was simultaneously conducted whilst retaining its independence. The BT was solely responsible for producing impactful findings that aimed at supporting the identification of ethically relevant aspects of the trial process and practice. This included identifying ethical issues raised by study participants and bringing these to the attention of the TT so that issues could be appropriately addressed. This approach utilized a range of qualitative research methods [30] which were embedded in the ethics study’s longitudinal data collection functions.

### The study setting

This study’s fieldwork was conducted in seven villages in Kasungu district, Central Region of Malawi. The area is predominantly characterized by subsistence farming alongside smallholder and estate tobacco production.

### Sample and sampling

In this study, women and their male partners were conveniently sampled to participate in the focus group discussions (FGDs). We approached study participants that were available for interviews on the set date. Additionally, village chiefs, health surveillance assistants, religious leaders, health volunteers, and agriculture volunteers were purposively sampled to participate in the in-depth interviews (IDIs) focusing on their social interaction status, and ensuring a diverse set of roles and responsibilities relevant to the trial, including health, agriculture, and community welfare. Table 1 provides the number of participants in FGDs and IDIs.

**Table 1:** Number of study participants who participated in FGDs and IDIs

Data collection method	Type of participants	Phase 1(# of participants)		Phase 2 (# of participants)		Total # of participants
		Male	Female	Male	Female	
In-Depth Interviews	Village chiefs, health surveillance assistants, religious leaders, health volunteers, and agriculture volunteers	2	8	2	8	20
Focus Group Discussions. (n groups = 14). Range 6–10 participants per group	Female trial participants and their male partners	27	35	33	35	130
Total		29	43	35	43	150

### Data collection

Data were collected using three approaches: observations, IDIs and FGDs. To enhance the validity of the study findings, data from the three approaches were triangulated [31]. This also assisted the researchers to write detailed ethnographic notes and record the participants’ perspectives.

### Initial interviews

We conducted seven FGDs each lasting 60 min and ten IDIs each lasting c.40 min during Phase 1. The questions and conversations were designed to draw out descriptions of trial participants’ (TPs) experiences. Open-ended and descriptive questions (e.g., what are your experiences in the trial?) were used in the FGDs and IDIs. Interviews were conducted in places that were convenient to trial participants such as community clinics, church centers, flour distribution centers, primary schools, and trial participants’ homes. These data were collected by two researchers with professional backgrounds in bioethics and qualitative research. The two researchers conducted the IDIs and FGDs in Chichewa, which is Malawi’s national language. Information sheets were shared with all potential participants for their comprehension of the study and participants were asked to give their informed consent to this work, outside of their participation in the AHHA trial, indicating they understood the study specifics and their willingness to voluntarily participate. Participants were reminded of their right to withdraw and that their decision would not jeopardize their involvement in the AHHA trial.

### Observations

#### Passive: non-participant observations

A total of four purposively sampled non-participant observations were conducted during key community sensitisation and trial activities. The first sensitisation meeting was conducted in April 2019 at LUANAR maize fields in Lilongwe. Kasungu is approximately 130 km northwest of the capital of Malawi, Lilongwe. The second meeting was conducted in Kasungu in preparation for the flour distribution exercise and the third visit was at the flour processing unit at LUANAR in Lilongwe, both in July. The last meeting was at the endline sensitisation meeting that was conducted in August 2019. The observations provided the researchers with important insights about the interactive experiences that were widely performed by both the TT and participants as they unfolded during the AHHA trial [32]. In this study, the units of observations were the participants and the activities that informed the theoretical sampling. Data was recorded by ethnographic field notes, which included a diary that was used by the researchers throughout the study.

#### Participant observations

The BT researchers actively participated in one meeting, the community dish sharing gathering. The approach permitted the BT to be directly involved with participants as they experienced cooking and eating “nsima” (Malawi’s staple food) made from the trial maize flour. This activity involved observing behavior, interaction with participants, and further exploration of participants’ perspectives and experiences. Throughout this experience, the knowledge and understanding of participants’ trial experiences evolved. In this study, observations were more effective when they were conducted alongside FGD and IDIs that were utilized to explore underlying reasons for trial activities, social interactions, and community behavior.

### Theoretical sampling and ongoing data analysis and alteration of interviewing route

Grounded Theory Methods are categorised by theoretical sampling [33] which is the purposive selection of a sample that allows further investigation of emerging categories. After Phase 1, detailed life experiences of the participants and their social interactions emerged. The study unearthed participants’ concerns that included the incidence of rumours and misconceptions. Therefore, open-ended questions were asked (e.g., How willing were participants to consume the given flour despite rumours about the trial?). The aim was to understand what would support the participant and identify issues that may need addressing by the TT. For this reason, we theoretically sampled Phase 1 participants for Phase 2 data collection. Therefore, in Phase 2, categories were “coping strategies” and strategies employed by the TT, which were initiated in response to any negative trial experiences as they were identified. In total, 14 FGDs and 20 IDIs were completed in this study.

### Data analysis

#### Memo writing

Memos were extensively written throughout the study after every observation and interview conducted [34]. The researchers held discussions reflecting on social interactions, the behaviour of participants, and the identification of new themes. Reflections from memos guided the BT in developing questions for the next interviews.

#### Coding and constant comparative methods

The data were arranged in chronological order and this enhanced the examination of the whole research process. Systematic data coding [35] was used, and open coding, which involved an analytic process in grounded theory methods in which concepts (codes) to the observed data and phenomenon are attached during qualitative data analysis [34]. This was followed by axial coding in which the researcher mapped connections or relationships between the various codes, which were identified during open coding, to group them into categories [33]. Lastly, the researcher conducted selective coding to integrate and develop the core categories which represented the central thesis of this study. The data set was managed using NVivo 12.0 software.

### Quality assurance

This study employed various techniques at each stage to achieve credibility, dependability, conformability, and transferability [36]. To enhance the credibility of the study findings, the research team kept comprehensive notes of the study through memos. The interviews and data analysis reports were widely discussed with the TT to reach a common understanding. Using the comparative methods [37] assisted the analysis to produce a model for explaining the social process of the study. The research team has presented a detailed report of the research process, from the data collection phases to the data analysis and interpretation phases, to enhance the study’s dependability.

To enhance confirmability of the study findings, the research team analysed the collected data and developed a research report that was shared with the AHHA TT after Phases 1 and 2 of the data collection process.

### Ethics approval and ethical issues

The study obtained its ethical approval from the College of Medicine Research Ethics Committee (reference number P.03/19/2633). Ethical approval for the AHHA trial was obtained from the College of Medicine Research Ethics Committee (reference number P.11/18/2539) and the LSHTM Interventions Research Ethics Committee (reference number: 16181). We consented study participants to the first FGDs and IDIs and they were notified of the arrangements for the second phase of data collection. All information was securely stored in password-protected files.

## Findings

The findings highlight the main categories of participants’ experiences and motivation to adhere to study requirements as identified through our analysis. The key categories identified in this study are summarised below, with stages in the participants’ trial experiences as follows:

- Seeking hope, dealing with contextual factors.
- Trial protocols: Acknowledging challenging experiences as a trial participant
- Adhering to trial requirements: Using coping strategies.
- Mastering the experience, finding value and hope.

Cutting across these temporal aspects were the participants’ experiences of interacting with the TT and the teams' responses to their questions, needs, and concerns. These are set out under the theme of the ‘value of the participant-TT deliberative processes.

### Seeking hope, dealing with contextual factors

Many TPs revealed they were unable to meet reasonable basic living expenses such as food and clean water. Thus, being in economic hardship was seen as a hopeless situation affecting the well-being of the community. The majority of the TPs equated the trial as a “*life-saving” initiative* (FGD 301 P1) as they perceived their economic issues will be resolved in the short term. This is illustrated by FGD 201, P1: *“But if you know that you are amid food scarcity – hunger, and you are sleeping hungry so when you hear that they are coming here, the flour people… you cannot refuse.”*

These comments are typical of what most TPs expressed in Phase 1 before the flour was distributed. The trial intervention was seen to offer hope as TPs were optimistic they will overcome everyday challenges by enrolling in the trial.

Participants discussed their understanding of what research is all about. It was seen to facilitate TPs’ expectation of how the findings of the trial would uplift their lives, for example, *“finding things that can help us”;* “*this is the first big research of this kind to happen”*, as elucidated by FGD 201*,* P1*.* It was clear that prior research engagements as evidenced here *“research on how to make manure”; “research about sanitation and hygiene”* FGD 203, P1 brought hope and higher expectations to TPs that the new research project would address their basic needs concerns. This is also illustrated here; “*research to develop this area”; “our main problem in this area is clean portable water”; “research should benefit the research area”* FDG, 201 P1. In the early stages of data collection, TPs expressed a strong view that research projects should bring a benefit to the community, and their motivation to adhere to trial requirements was a significant part of dealing with contextual issues.

The majority of participants equated being randomised in the trial as being lucky as they perceived trial participation was an honour. Thus, being randomised was interpreted as a great opportunity that they would gain something desirable as stated by e.g., P10, P2, indicated they were “*lucky and having a great chance”.* These comments were typical of what most trial participants expressed in the second phase of the data collection exercise. Most participants expressed that by fully adhering to study requirements, they were very hopeful something of good fortune would follow them.

Many TPs revealed that in the past they have been deceived by people who claimed to be from Non-Governmental Organisations therefore the engagement of key gatekeepers was seen as a way of dealing with issues of mistrust and social structure. The involvement of key gatekeepers gave hope as it signalled the trial was for a good cause as evidenced by *“We should receive it. Because [our] grandfather, Traditional Authority Wimbe or the government, cannot allow bad things to happen to us*” Male Volunteer, IDI 105, P1.

Dealing with the contextual issues was therefore seen to offer hope that motivated TPs to take up their trial roles and responsibilities. TPs were willing to adhere to trial requirements only when their present contextual situation was more bearable but also if they perceived that their involvement would eventually improve their lives. TPs had hoped that the outcome of the trial would make their life better in some way.

### Trial protocols: acknowledging challenging experiences as a trial participant

Many acknowledged dealing with challenging experiences as they adhered to the trial protocol. TPs revealed they perceived the significance of adhering to all trial procedures however, rumours, myths, jealousy, and misconceptions created feelings of fear and anxiety. Many TPs echoed that they experienced social harm due to the withdrawal of their blood at baseline. Community members called them by various names like “*kudya ufa wamagazi*” (eating bloody flour), “*magulitsa magazi*”(selling blood), “*opereka magazi”* (blood donors), and rumours were present as illustrated by FGD 203, P1:

*“They are taking your blood so that they can sell. And also, all those whose blood was taken will die.”*

During phase 1, it was evident that many individuals felt the burden of being a TP. Reflecting on the arrangement that every household was receiving freely-distributed flour whether or not they were donating blood was frustrating for some. This was illustrated in FGD 304, P2:

*But those who did not give blood their flour stays until they receive again. Maybe they are even the ones receiving a lot of flour. So, they say what is the gain when we gave blood but receive little flour, they did not but receive a lot of flour. What did we gain by giving blood?*

Many participants acknowledged that beliefs and rumours about blood were widespread and continued to occur among community members during the trial period. Rumours about blood included blood being perceived as sacred:

*The donated blood would be used for satanic rituals; those who donated blood would have fertility issues especially blood donors and their partners* (IDI 302 P1)*.*

*Blood donors would swell and die and donated blood would be sold* (FGD 304 P1)*.*

Many participants articulated the experience of dealing with various trial misconceptions. Humanitarian or philanthropic misconceptions were triggered by expectations among participants and community members, misinformation, and lack of understanding of trial activities, procedures, and goals of research, as illustrated by FGD 305 P2’s verbatim response: *“That is where I said the research is good because if someone is giving you flour, that means you are saving our lives”.*

Many TPs articulated that community members had challenges in understanding the goals of the trial hence found it difficult to ascertain why trial procedures had to be adhered to. Lack of understanding by those not participating in the trial accelerated confusion, fear, and anxiety. Soon after flour distribution, it became evident that some trial requirements had an impact on TPs’ social relationships. It was revealed that TPs were ridiculed for refusing to share or to eat non-trial flour during weddings and funerals. This is evidenced by FGD 304, P2:

*They asked us: Why are you eating your flour on your own? So, we said your flour and ours are different, that is why you can eat yours anyhow. Those who gave blood were told not to mix the flour. So, they then said that no matter what procedure we follow or if we gain weight, the Bunda [LUANAR] folks will not give us cars, the cars are theirs. We quarrelled because of this issue; men had to stop us.*

Signing up to be involved in the trial reflects various changes and challenges that emerged as TPs consent to adhere to trial requirements. TPs experienced some changes in their social relationships and everyday life due to personal commitment and responsibilities to the trial.

### Adhering to trial requirements: using coping strategies

The TPs who acknowledged dealing with challenging experiences reported several coping strategies that helped them develop self-efficacy. The absence of adverse effects gave TPs the courage to start offsetting rumours and misconceptions. Fertility rumours were of great concern amongst TPs and their partners. To identify if there were issues with the flour, TPs and their partners reportedly observed their sexual performance to identify any changes or concerns. This is evidenced through FGD 305 P2 who said *“Now, we are even conceiving more since we are not going to bed hungry, everybody is eating. But what they were saying is not happening. Because those of us who gave blood are just brave”.* As they identified no issues, TPs became very determined to commit to trial roles and even took up a role to educate fellow community members.

During the second phase of the data collection exercise, it was clear that TPs expressed a strong sense that they were very significant to the research project and the community at large. TPs’ attitudes towards the trial changed as they achieved a sense of mastery and community members started seeking validation about the trial from them. They were being referred to as the *“special group”.* Their image as those who had sold their blood was reconstructed to something positive as they began attaching positive labels to their role and identity. Some of these labels include *“the chosen ones” (“osankhidwa”*, in vernacular) (FGD 305 P2) and *“owners of the programme” (“eni pologramu”,* in vernacular) (FGD 305 P2). The change in social status triggered new perspectives on benefits and rewards.

It became evident in the second phase that TPs who shared their lived experiences increased motivation to adhere to trial requirements. TPs reflected on various strategies that were employed by the TT and it was widely regarded as the TT role to protect TP welfare. The TT intervened and responded to the TPs’ concerns at various time points. The TT response appears to be an important temporal aspect in terms of the participants’ concerns, need to discuss and then feelings of support and finally finding a reflective approach and even pride at the end of the study. Participants demonstrated a need for continuous support, which appeared to help to reduce concerns over time. These findings are examined below as the value of the TP and TT deliberative process.

### The value of the participant-trial team deliberative process

Participants reflected on some of the important features that motivated them to remain within the trial and to continue with the protocol. These include the strategic communications provide by the TT and the generation of relevant information throughout the trial. They also include the willingness and ability of the researchers to provide more information about the trial, as demanded by participants. During the trial, beyond the initial consent process, participants had opportunities to ask questions and share their trial experiences, including the interactive communications between the TT and TPs. The TT conducted timely activities that were perceived to help TPs confidence levels as narrated by FGD 201 P1;

*“Mainly, this relationship can teach everything that needs to be taught... When they asked questions, they would be answered accordingly. That is what mainly made the relationship work because we were able to understand”.*

Participants revealed how ongoing sensitisation meetings and education sessions supported the acquisition of new and relevant knowledge at different times resulting in enhanced comprehension of the trial requirements, this is reflected by a focus group participant (FGD 305 P2):

“*There is nothing that can disturb it because the supervisors came the day before yesterday. They explained well the procedure they followed which removed everyone's doubts, so we are not going back. Before every procedure happens, we are educated or sensitised. That is why this research is going on well until the end”*

Participants also valued various activities that were implemented by the TT (Table 2).

**Table 2:** AHHA trial interactive sessions with the communities and trial participants period

Period (2019)	Activity	Objective
April	LUANAR Maize field tour	Kasungu Community members visited LUANAR farms to understand how the intervention maize was grown, understand how Selenium fertilizer techniques were applied. An opportunity to discuss with the LUANAR team about overall maize and study-related information
July	Sensitisation Meeting (After Baseline Study and Ethics FGD and IDI)	Meeting organized by the trial Implementing team in a trial community area
		These meetings were scheduled in response to the ethics engagement exercise: The following were topics of discussion
		Safety of flour and flour consumption procedures
		To refute rumors about blood donation
		To highlight milestones on study activities and a reminder on study roles and responsibilities
		Discuss flour distribution procedures
		Discuss information needs, answer questions
July	LUANAR Flour Processing visit	Kasungu Community members visited LUANAR for an opportunity to visit the maize mill where dehulling and determining of the maize grain and maize flour milling was taking place
July	Community dish sharing activity	This was a dish-sharing event that was organized by the LUANAR team to cook and eat the study flour together with participating village members, Kasungu district key stakeholders before the trial flour consumption commenced. It was organized as an event to nullify rumors, alleviate fear about the flour, and cement trust
September	Endline Community Information Sharing Sessions	These meetings were scheduled in response to the ethics engagement exercise: The following were topics of discussion:
		Highlight the importance of endline blood donation
		Motivate study participants to donate blood and adhere to flour consumption
		Discuss blood donation procedures
		Discuss information needs, answer questions and hear concerns and fears

Many participants commended the TT for their transparency and commitment to enhance positive experiences, exemplified by these comments:

*People were now encouraged that there was a demonstration on cooking the flour. We ate it in the group* (FGD 302 P2).

*So it was encouraging the people because they made a program to see the food and also the food should be cooked we should all eat. And visiting those people who gave blood also encouraged” FGD 301, P2; ‘The first time we went to Bunda because we went twice, we saw different types of maize in the field with the fertiliser that was applied.”* (IDI 304 P2)*.*

Many participants expressed support after careful consideration of the TT’s response to their concerns, such as emotions, uncertainties, fear of exploitation, and anxiety. Participants described opportunities to discuss rumours, misconceptions, and myths as an opportunity to further understand and acquire more knowledge about the trial activities at that time point. The knowledge assisted them in refuting rumours and misconceptions, as reflected by Male Volunteer, IDI 102 P1:

*They should be coming every now and then, teaching those people that are left behind to join the other group because some were left behind indeed, they did not understand. So we were thinking that these meetings should continue happening now and then up to the time we are about to receive, so that those people should be taught*”

Participants perceived that the TT had implemented an approach that created “conversation spaces” to listen to their concerns and accept their suggestions. It became very evident that the TT listened to the TPs’ information needs.

### Mastering the experience, finding value and hope

Participants expressed a form of mastery of their trial experiences, which might be called ‘finding value and hope’. Participants reported various coping strategies described above which promoted their self-efficacy and decision-making abilities. Many participants integrated the various perceptions that emerged multiple times throughout the trial and used current and past experiences and information to make decisions to support their continued involvement in the trial. They shaped new forms of hope and value by requesting from the TT continuous information on how the expected improvements from the intervention might benefit their communities, with some holding high expectations to learn from the trial results, as reflected by a male participant in the focus group (FGD 302 P2):

*“That is why we have taken ownership and can leave the flour to the wife [narration of a male partner] because she is the one whose blood was tested, and a time is coming when she will be tested. We are also just grateful that the wife has not eaten the food we grow ever since she gave blood. We really want her to be eating this food until the research ends so that when giving the results, we do not cheat you. Because the research would be useless if we do not play a part*.

This position was also shared by a Female Participant (IDI 110, P2):

*The role I took was to heed the part I have been assigned as I joined the research. I should follow what I was advised to do so that at the end I should see the results.*

Several participants questioned how negative findings would be managed by the TT despite them complying with trial requirements (IDI 102, P2):

*What touches me on this research is that, as I am eating this flour, so when testing me what will happen if you find that what you wanted was not achieved? But I am eating the flour the way you told us*.

Participants expressed being able to request information from the TT to help them establish strategies that would protect them from wider social harm. For many TPs, a responsive TT was very critical in helping them achieve self-efficiency. TPs were likely to refute rumours and misconceptions if they had accurate and relevant information about the trial at various time points and a responsive TT would address their concerns according to their context and concerns to promote/mitigate social harm.

## Discussion

Participants’ prior knowledge and experiences were critical on how this research was interpreted and how research activities were understood. This is a similar finding to a study conducted by Meade [38], who argued that adults are not a ‘blank slate’ and prior knowledge is critical in enhancing the comprehension of new knowledge [38]. Many TPs demonstrated knowledge about the objectives and clearly outlined trial activities. However, at the start of the study, TPs narrated various expectations outside the scope of the trial ranging from essential services that included the provision of clean potable water, bridges, farm inputs, and hospitals. This could be attributed to poor social-economic conditions in their respective communities and the issues associated with a humanitarian or philanthropic misconception that was revealed among TPs. A report by Wang, revealed that philanthropic misconception is related to a notion of trust arising when recipients entrust those who exhibit goodwill or benevolence towards them, thereby misunderstanding the purpose of the activity, while overrating the intended benefits or underestimating potential risk [39]. In the context of this study, there were two sets of anticipated benefits, direct benefits to those who participated in the trial and long-term benefits towards the welfare of the overall communities. The study also revealed interesting findings on how philanthropic misconceptions may have introduced or exacerbated existing or new vulnerabilities among TPs. It was reported that if TPs failed to satisfy the objectives of the study and researchers failed to meet their benefit expectation, this could negatively affect their social and community relations making the TPs vulnerable to social harm [40].

This study revealed how challenging it is to achieve voluntary participation in low-resource community settings, as structural coercion can define perspectives that affect the decision to enroll in research [35, 36]. This study highlights that some of the participants expressed good fortune to be included in the study, implying an unreflective socio-economic influence on their participation in the study. Findings from this study resonate well with Nyirenda’s [41] findings in Malawi, which stated that the socio-economic context and unequal power relations influence decision-making rendering participants vulnerable to exploitation. The findings, therefore, highlight the need for RECs to focus on the risks of undue influence and preventing exploitation especially in settings with underlying contextual factors. The findings from this study are in line with the findings from a study done by Fisher [42] which recommended various strategies for addressing structural cohesion which proposed RECs should apply a more robust analysis to assess structural conditions that may affect voluntary participation. The findings here also imply that RECs should more closely examine what supporting and deliberative mechanisms are put in place by Trial Teams across the life of trials. Extending this work, it will be informative to compare the current study findings drawn from a community-based trial with participant experiences from clinical settings [43]

In the initial phase of the study, participants experienced emotions including fear, anxiety, uncertainty, and stress related to “rumours about blood selling and stealing”, safety concerns, myths, and misconceptions. Participants reported negative experiences related to psychological distress that was associated with randomisation and trial activities. This is consistent with previous studies conducted in low- and middle-income country settings that indicate underlying negative trial experiences are commonplace, with historic and socio-economic factors contributing to concerns [23, 44, 45]. In this study, the issue of “blood stealing and selling” was ever present, highlighting how widespread misconceptions about blood are in some contexts in Africa [22, 24, 46, 47].

This study has shown that researchers require strategies to determine the diverse nature of risk for participants, the social acceptability of studies, and what rumors and types of fear may shape participation narratives for both pre-and post-consent individuals [45]. Participants also expressed the notion of burden, revealing how principles of the trial design in community settings influence various narratives about risks. It could be argued that at times participants expressed a notion of sacrifice as part of their motivation, as they struggled for self-reliance and social recognition [48]

Mapping and reporting back to the TT about participants’ concerns and their narratives of risk perception enabled the TT to respond to and put in place approaches to empower participants, [49] build partnership [50], enhance the informed consent and continuous consent process [51] resulting into improved self-efficacy [52]. The efforts of participants to develop self-efficacy were shared through narratives based on self-motivation and behaviour. Features of community social structures, socio-economic status, and principles of trial design motivate participants to commit to a research study. However, participants took their role seriously and appeared to develop self-efficacy beliefs over time that influenced task choice, determination, persistence, resilience, and achievement [53]. In addition, participants championed their learning and mastery of activities revealing how information seeking and beliefs greatly improved over time. Many of the approaches that were provided by the TT were embedded in various social networks within the communities, hence these support approaches assisted them to succeed. The findings of this study suggest that researchers require embedded ethical strategies that have temporal sensitivity and directly gauge what the community and researchers identify as social and ethical issues, as well as using traditional ethical guidance and expert opinion [54].

This study shows that participants valued collaborative engagement with the TT. Opportunities to express their fears and concerns, verify information, and participate in various trial information-sharing activities were reported as some of the strategies that enhanced the informed initial and continuous consent process. The emphasis on partnership building, empowerment, and collaborative engagement supports the claim that researchers need to interact with participants and discuss the realities of participating in trials where there may be issues that can affect adherence to study protocols and even retention [55].

The motivation that participants had to know about the outcome of the trial may be perceived by researchers to be less relevant in poor resource settings like Kasungu, where the socio-economic status of communities is low. However, this motivation and sense of ownership of the trial may take on a different meaning is seen as a way to demonstrate the value of participation and lessen the notion of social harm within a community setting. One of the limitations of this study was that study participants’ perspectives on the notion of exploitation, taking into consideration how participants overcome negative experiences, was not explored in depth. This would be an important aspect of a future study.

This study suggests that participants’ self-efficacy does not come from simply setting protocols and instructing participants to follow these, but is rather a responsibility for researchers to consider how they develop messaging and engagement strategies that can respond to local contexts and participants' needs [56]. In this study, self-efficacy included being able to offset myths and misconceptions about the flour, suggestions, and clues to food preparation, strategies to identify and report adverse events, as well as strategies to share information with others, to seek more information, ask questions, share emotions and concerns.

In community contexts characterised by widespread poverty as in Malawi, issues of mistrust [57], rumours and fear about clinical research [58], power inequalities [59], respect for persons, reciprocity [45], altruism [60] and hope are all important elements that can affect participants’ willingness to take part and experiences within studies. This is corroborated by those studies that have been conducted in low- and middle-income settings outlining some of these factors as motivating for study participation [48]. In this study, participants expressed concerns yet they also expressed their commitment as underpinned by their social value expectations [48] and highlighted the personal value of the TT deliberative approach to their lived experiences.

## Conclusion and recommendations

The findings of this study reveal that a willingness to consent to be part of a research project can hide a wide range of initial and ongoing concerns amongst study participants, which highlight the need to further protect participants’ rights and support those who consent through the life of a project. This work shows that taking part in a community-based trial can be challenging for individuals in a low resource setting, as committing to the role of a trial participant can raise ethical and social issues due to the disruption of people’s everyday life and the impact of participation can have on social interactions within a community. This work highlights the influence of contextual factors and social and economic status on negative experiences in the early stages of a trial. However, the processes of mapping and sharing participants’ ethically-relevant concerns which are combined with a series of responsive approaches from the trial team during the study, can support participants as they identify the purpose in their participation, help them create coping mechanisms, and re-framed their trial experiences. This further led to a form of perceived ownership of the trial with participants emphasising to the researchers the importance of sharing the trial results directly with them, sharing experiences, and working through their concerns. This offers great insights on the need to develop and apply strategies to support participants and to enhance their positive trial experiences.

The findings offer important lessons for the value of exploring ethical and social issues in real-time during a study. Leveraging from empirical ethics in community research in low resource settings, this study has revealed how principles of study design, social structures, and social and economic contexts contribute to diverse ethically relevant perspectives on trial participation. Further research to improve the practical application of informed consent and how ongoing participant support and embedded fulfilment of ethical principles can be achieved which are also tailored to local contexts is needed to enhance ethical conduct of research and enhance uptake of study roles and responsibilities. The importance of these issues is demonstrated in this work and more widely, it is therefore key that going forward researchers employ embedded ethics strategies that are focused on empowering study participants through enhanced informed consent processes. We propose that researchers develop ethical engagement plans in the same way that data management plans are now ubiquitous and to support researchers’ efforts, we propose that RECs thoroughly review informed consent and engagement plans to assess structural conditions that may affect voluntary participation.

## Data Availability

The datasets generated and/or analysed during the current study are not publicly available because permission to share transcripts and all data generated during this study outside of the GeoNutrition ethics project team was not sorted from study participants but are available from the corresponding author on reasonable request.

## Abbreviations

COMREC: College of Medicine Research Ethics Committee
FGD: Focus group discussion
IDI: In-depth interview
KI: Key informant
REC: Research Ethics Committee
BT: Bioethics team
TPs: Trial participants
RTREA: Real-time research ethics approach
AHHA: Addressing hidden hunger with agronomy
GMT: Ground theory method (GTM)

## References

[1] Zheng W, Chang B, Chen J (2014). Improving participant adherence in clinical research of traditional chinese medicine. Evid Based Complement Alternat Med..

[2] Council for International Organizations of Medical Sciences (CIOMS). International ethical guidelines for biomedical research involving human subjects. Guideline. 2016 [Internet]. 2016. Available from: https://cioms.ch/. 14983848

[3] Vreeman R, Kamaara E, Kamanda A, Ayuku D, Nyandiko W, Atwoli L (2012). A qualitative study using traditional community assemblies to investigate community perspectives on informed consent and research participation in western Kenya. Med Ethics.

[4] Kaye DK (2021). Motivation to participate and experiences of the informed consent process for randomized clinical trials in emergency obstetric care in Uganda. BMC Med Ethics.

[5] McCann SK, Campbell MK, Entwistle VA (2010). Reasons for participating in randomised controlled trials: conditional altruism and considerations for self. Trials.

[6] Tom L, Beauchamp JFC (2001). Principles of biomedical ethics.

[7] Nishimura A, Carey J, Erwin PJ, Tilburt JC, Murad MH, Mccormick JB (2013). Improving understanding in the research informed consent process : a systematic review of 54 interventions tested in randomized control trials. BMC Med Ethics.

[8] Dixon-Woods M, Ashcroft RE, Jackson CJ, Tobin MD, Kivits J, Burton PR (2007). Beyond ‘“ misunderstanding ”’: written information and decisions about taking part in a genetic epidemiology study. Soc Sci Med.

[9] Haynes RB, Dantes R (1987). Patient compliance and the conduct and interpretation of therapeutic trials. Control Clin Trials.

[10] Susilo AP, Marjadi B, van Dalen J, Scherpbier A (2019). Patients’ decision-making in the informed consent process in a hierarchical and communal culture. Asia Pacific Sch.

[11] Claramita M, Susilo AP (2014). Improving communication skills in the Southeast Asian health care context. Perspect Med Educ.

[12] Hughson J, Woodward-Kron R, Parker A, Hajek J, Bresin A, Knoch U (2016). A review of approaches to improve participation of culturally and linguistically diverse populations in clinical trials. Trials.

[13] Tam NT, Huy T, Bich T, Long P, Huyen T (2014). Participants’ understanding of informed consent in clinical trials over three decades: systematic review and meta-analysis. Bull World Health Organ.

[14] Kennedy BM, Harsha DW, Bookman EB, Hill YR, Rankinen T, Rodarte RQ (2011). Challenges to recruitment and retention of African Americans in the gene-environment trial of response to dietary interventions (GET READI) for heart health. Health Educ Res.

[15] Breckenridge A, Aronson JK, Blaschke TF, Hartman D, Peck CC, Vrijens B (2017). Poor medication adherence in clinical trials: consequences and solutions. Nat Rev Drug Discov.

[16] Mfutso-Bengo J, Ndebele P, Jumbe V, Mkunthi M, Masiye F, Molyneux S (2008). Why do individuals agree to enrol in clinical trials? A qualitative study of health research participation in Blantyre, Malawi. Malawi Med J.

[17] Alemayehu C, Mitchell G, Nikles J (2018). Barriers for conducting clinical trials in developing countries—a systematic review. Int J Equity Health.

[18] Kadam R, Borde S, Madas S, Salvi S, Limaye S (2016). Challenges in recruitment and retention of clinical trial subjects. Perspect Clin Res.

[19] Chukwuneke FN. The challenges in enrolment and retention of African women in clinical trials: a pilot study in Nigeria. J Clin Res Bioeth. 2012;03(01):1–3.

[20] Weaver CM, Miller JW (2017). Challenges in conducting clinical nutrition research. Nutr Rev.

[21] Joy EJM, Kalimbira AA, Gashu D, Ferguson EL, Sturgess J, Dangour AD (2019). Can selenium deficiency in Malawi be alleviated through consumption of agro-biofortified maize flour? Study protocol for a randomised, double-blind, controlled trial. Trials.

[22] Boahen O, Owusu-Agyei S, Febir LG, Tawiah C, Tawiah T, Afari S (2013). Community perception and beliefs about blood draw for clinical research in ghana. Trans R Soc Trop Med Hyg.

[23] Kingori P, Muchimba M, Sikateyo B (2010). 'Rumours' and clinical trials: a retrospective examination of a paediatric malnutrition study in Zambia, southern Africa. BMC Public Health.

[24] Compaoré A, Dierickx S, Jaiteh F, Nahum A, Francis T, Bohissou E (2018). Fear and rumours regarding placental biopsies in a malaria-in-pregnancy trial in Benin. Malar J.

[25] Manuscript A (2014). Participants’ responsibilities in clinical research. J Med Ethics.

[26] Marshall PA (2007). Ethical challenges in study design and informed consent for health research in resource-poor settings WHO Library Cataloguing-in-Publication Data Ethical challenges in study design and informed consent for health research in resource-poor settings. WHO Libr Cat.

[27] Dada S, McKay G, Mateus A, Lees S (2019). Lessons learned from engaging communities for Ebola vaccine trials in Sierra Leone: reciprocity, relatability, relationships and respect (the four R’s). BMC Public Health.

[28] Chiutsi-Phiri G, Kalimbira AA, Banda L, Nalivata PC, Sanuka M, Kalumikiza Z, Joy EJ. Preparing for a community-based agriculture-to-nutrition trial in rural malawi: findings from formative research. https://pilotfeasibilitystudies.biomedcentral.com/. 2019. 10.1186/s40814-021-00877-1 PMC8262007 34233757

[29] Ulin PR, Robinson ET (2005). Qualitative methods in public health.

[30] Blanche MT, Durrheim K. Research in practice: applied methods for the social sciences, 2nd ed. In: Blanche MT, Durrheim K, editors. University of Capr Town; 2006. p. 587.

[31] Thurmond VA (2001). The point of triangulation. J Nurs Scholarsh.

[32] Shope R, Creswell JW, Shope R, Clark VLP, Green DO (2006). How interpretive qualitative research extends mixed methods research. Res Sch.

[33] Charmaz K, Holstein JA, Gubrium JF (2008). Constructionism and the Grounded Theory. Handbook of Constructionist Research.

[34] Sbaraini A, Carter SM, Evans R, Blinkhorn A (2011). How to do a grounded theory study: a worked example of a study of dental practices. BMC Med Res Methodol.

[35] Chametzky B, College J (2016). Coding in classic grounded theory: i’ ve done an interview; now what ?. Social Mind.

[36] Patton MQ (1990). Enhancing the quality and credibility of qualitative analysis. Health Service Method.

[37] Mack N, Woodsong C, MacQueen K, Guest G, Namey E (2005). Qualitative Research Methods: A Data Collector’s Field Guide.

[38] Meade CD (1999). Improving understanding of the informed consent process and document. Semin Oncol Nurs.

[39] Wang N (2020). “We live on hope…”: ethical considerations of humanitarian use of drones in post-disaster Nepal. IEEE Technol Soc Mag.

[40] Milford C, Barsdorf N, Kafaar Z (2007). What should South African HIV vaccine trials do about social harms?. AIDS Care Psychol Socio-Med Asp AIDS/HIV.

[41] Nyirenda D, Sariola S, Kingori P, Squire B, Bandawe C, Parker M (2020). Structural coercion in the context of community engagement in global health research conducted in a low resource setting in Africa. BMC Med Ethics.

[42] Fisher JA, Fisher JA (2015). Expanding the frame of “voluntariness” in informed consent: structural coersion and the power of social and economic context. Kennedy Inst Ethics J.

[43] Lawrence DS, Tsholo K, Ssali A, Mupambireyi Z, Hoddinott G, Nyirenda D (2021). The lived experience of participants in an African RandomiseD trial (LEOPARD): protocol for an in-depth qualitative study within a multisite randomised controlled trial for HIV-associated cryptococcal meningitis. BMJ Open.

[44] Kerr C, Robinson E, Stevens A (2004). Randomisation in trials: do potential trial participants understand it and find it acceptable?. J Med Ethics.

[45] Ndebele PM. A study of trial participants’ understanding and attitudes towards randomisation, double-blinding and placebo use, and a pilot intervention in a microbicide trial in Malawi. 2010;501. Available from: http://citeseerx.ist.psu.edu/viewdoc/download?doi=10.1.1.824.5694&rep=rep1&type=pdf.

[46] Enawgaw B, Yalew A, Shiferaw E (2019). Blood donors’ knowledge and attitude towards blood donation at North Gondar district blood bank, Northwest Ethiopia: a cross-sectional study. BMC Res Notes.

[47] Grietens KP, Ribera JM, Erhart A, Hoibak S, Ravinetto RM, Gryseels C (2014). Perspective piece: doctors and vampires in Sub-Saharan Africa: ethical challenges in clinical trial research. Am J Trop Med Hyg.

[48] Fayia A, Enria L, Smout E, Mooney T, Callaghan M, Ishola D (2018). Social science & medicine “ we are the heroes because we are ready to die for this country ” : participants ’ decision-making and grounded ethics in an Ebola vaccine clinical trial. Soc Sci Med.

[49] Cascio MA, Racine E (2018). Person-oriented research ethics : integrating relational and everyday ethics in research. Account Res.

[50] Rimal BRN (2001). Perceived risk and self-efficacy as motivators : understanding individuals ’ long-term use of health information. J Commun.

[51] Flory J, Emanuel E (2004). Interventions to improve research participants’ understanding in informed consent for research: a systematic review. JAMA..

[52] Turner MM, Rimal RN, Morrison D, Kim H (2006). The role of anxiety in seeking and retaining risk information : testing the risk perception attitude framework in two studies. Health Commun Res.

[53] Bandura A. Bandura_Sociallearningtheory.Pdf. General Learning Corporation. 1971.

[54] Matandika L, Millar K, Umar E, Joy E, Mfutso-Bengo J. Operationalising a real-time research ethics approach: supporting ethical mindfulness in agriculture-nutrition-health research in Malawi. (2021) (Unpublished) 10.1186/s12910-021-00740-1 PMC8748184 35012535

[55] Maiter S, Simich L, Jacobson N, Wise J (2008). Reciprocity: an ethic for community-based participatory action research. Action Res.

[56] Banks S, Armstrong A, Carter K, Graham H, Henry A, Holland T, et al. Everyday ethics in community-based participatory research. 2013;2041.

[57] Kapumba BM, Jambo K, Rylance J, Gmeiner M, Sambakunsi R, Parker M (2020). Stakeholder views on the acceptability of human infection studies in Malawi. BMC Med Ethics.

[58] Mfutso-Bengo J, Masiye F, Molyneux M, Ndebele P, Chilungo A (2008). Why do people refuse to take part in biomedical research studies? Evidence from a resource-poor area. Malawi Med J.

[59] Mfutso-Bengo J, Masiye F, Muula A (2008). Ethical challenges in conducting research in humanitarian crisis situations. Malawi Med J.

[60] Van Schalkwyk G, De Vries J, Moodley K (2012). “It’s for a good cause, isn’t it?”—Exploring views of South African TB research participants on sample storage and re-use. BMC Med Ethics.

